# Editorial: New therapeutic strategies against carbapenem-resistant gram-negative bacteria

**DOI:** 10.3389/fmicb.2024.1513900

**Published:** 2024-11-07

**Authors:** Maria Teresa Mascellino, Alessandra Oliva, Silpak Biswas, Giancarlo Ceccarelli

**Affiliations:** ^1^Department of Public Health and Infectious Diseases, Sapienza University of Rome, Rome, Italy; ^2^Department of Microbiology, School of Tropical Medicine, Kolkata, India

**Keywords:** antibiotic resistance, antibiotics, bacterial pathogen, carbapenem, β-lactamase, carbapenem resistance

Multidrug resistance in bacterial pathogens poses a threat to human health, and the emergence of carbapenem-resistant *Enterobacterales* (CRE) infections seriously affects population welfare. The emergence of carbapenem resistance is a major concern, especially for intensive care units (ICUs) and other at high-risk wards, that has led to serious consequences (Tamma et al., 2021). Detailed studies identifying the mechanisms leading to carbapenem resistance in bacteria may help overcome and manage this Research Topic (Mascellino et al., 2024).

CRE often carry multiple resistance genes that are able to propagate through both vertical and horizontal routes (Rumbo et al., 2011). These resistance elements limit treatment options and some patients require a longer duration of therapy requiring intensive care with increased toxicities if compared to patients infected with carbapenem-susceptible strains. Therefore, new alternative approaches are needed to combat the spread of antimicrobial resistance in bacteria among populations and to treat patients infected with life-threatening carbapenem-resistant gram-negative bacteria (Oliva et al., 2021; Tompkins and van Duin, 2021). The development of new classes of antibiotics could be a solution. However the director of World Health Organization (WHO) Dr. Tedros Adhanom Ghebreyesus said verbatim that “Since mid-2017, only 13 new antibiotics have been authorized, with just two representing a new chemical class and considered innovative.”

Molecular studies play an important role in understanding the mechanisms underlying bacterial resistance. For example, mobile colistin resistance genes (mcr-1 to mcr-10) and their variants have been identified in gram-negative bacteria which pose a new threat to the treatment of clinical infections. A new method using a multiplex TaqMan real-time PCR assay was developed to detect mobile colistin resistance genes. This method has high specificity, sensitivity, and reproducibility (Gong et al.).

A previous study examined the *in vitro* drug susceptibilities of *Klebsiella pneumoniae* strains producing New Delhi metallo-β-lactamases in Poland. Cefiderocol, eravacycline, tigecycline, ceftazidime/avibactam (CAZ/AVI) and aztreonam were found to be the most effective antibiotics, demonstrating CAZ/AVI plus aztreonam 100% *in vitro* sensitivity with the tested strains. Owing to the safety of both drugs and their cost-effectiveness, this therapy should be the first-line treatment for carbapenemase-producing *Enterobacterales* infections (Słabisz et al.).

Ginkgolic acid, derived from Ginko biloba extracts, was identified as a potent inhibitor against KPC-2 and found to have no toxic effects. The evolution of resistance genes has limited the clinical application of carbapenems. Therefore, the synergistic effect of ginkgolic acid and carbapenems, especially meropenem, could be interesting and valid in the fight against antimicrobial resistance, potentiating the killing effect of carbapenems on KPC-2-positive *Klebsiella pneumoniae*. Generally, plant extracts exhibit good efficacy against microorganisms, specifically by altering the functional groups of KPC-2 (Song et al.).

From a study conducted in Portugal, extensively drug-resistant *Pseudomonas aeruginosa* (PA) is a growing concern because of its resistance to most common antibiotics, including carbapenems, piperacillin-tazobactam, third- and fourth-generation cephalosporins, aminoglycosides, and fluoroquinolones. Only one case of non-susceptibility to colistin has been previously reported. Isolates were susceptible to ceftazidime-avibactam and ceftolozane-tazobactam in 71.5 and 77.5% of tested isolates, respectively. When a combination therapy is used, ceftazidime-avibactam plus colistin is preferred, which leads to a lower mortality rate in patients with PA infections (Mendes Pedro et al.).

A network meta-analysis from South Arabia included data from over 25 clinical trials and 5,034 individuals to investigate the antibiotic resistance trends and treatment outcomes of gram-negative infections. *Pseudomonas aeruginosa* and *Acinetobacter baumannii* turned out to be more resistant than *Enterobacterales*.

In China, resistance of *Escherichia coli, Pseudomonas aeruginosa*, and *Acinetobacter baumannii* was evaluated by comparing strains isolated in ICUs with those isolated from non-ICUs over a period of 10 years. Sensitivity rates to amikacin, carbapenems, and piperacillin/tazobactam were relatively high compared to high resistance rates to fluoroquinolones. The isolates from ICUs showed greater resistance than the non-ICUs strains (Shi and Xie).

The combination of antibiotics leads to more activity, especially the association between β-lactams and inhibitors of β-lactamases, such as avibactam. In a study performed in Belgium, the authors validated Gradient Diffusion Strips (GDS) focusing on an aztreonam–avibactam gradient. A double-disc synergy test (DDST) was used as a screening tool for the synergistic detection of aztreonam and avibactam. Aztreonam used in combination with ceftazidime-avibactam is considered an effective therapy for *Pseudomonas aeruginosa* an*d Stenotrophomonas maltophilia* (Verschelden et al.).

A recent italian study evaluated the effectiveness of imipenem/cilastatin/relebactam for the treatment of KPC-producing *Klebsiella pneumoniae* complex and difficult-to-treat resistant *Pseudomonas aeruginosa* infections in 10 patients. The successful and safe use of imipenem/relebactam, a newly available and promising treatment option, for the treatment of KPC-Klebsiella pneumoniae or difficult-to-treat resistant *Pseudomonas aeruginosa* complicated infections, has been reported. This preliminary clinical experience with imipenem/relebactam is a very attractive option compared to other combinations, especially in Italy, where the prevalence of difficult-to-treat resistant organisms is a significant concern (Leanza et al.).

Risk factors for CRE colonization also increase the possibility of subsequent infections in patients with hematological diseases. These findings suggest that septic shock increases mortality in CRE-infected hematological patients. Clinicians should try to prevent the early onset of infection and take measures to reduce mortality rates in these patients. From this research, it emerged that mortality risk factors are higher in patients with hematological diseases (Wang et al.).

Controlled trials are crucial for studying antimicrobial routes and administration methods. In Taiwan, continuous meropenem infusion is reportedly much better for microorganism eradication than traditional intermittent bolus strategies. This does not allow for bacterial death but specifically contributes bacterial eradication (Ai et al.).

The burden of antimicrobial resistance, which is a worldwide problem, should be accurately examined, especially during the SARS-CoV-2 pandemic in ICUs. Italian researchers underline this Research Topic, which poses therapeutic challenges by providing valuable insights into the epidemiology of hospital infections. In view of the possibility of a future pandemic, this approach may lead to greater infection control (Scaglione et al.).

The combination of amikacin, polymyxin-B, and sulbactam demonstrated *in vitro* synergy against multidrug-resistant *Acinetobacter baumannii*. The approach was initially developed for adults and subsequently scaled for children. Pharmacokinetic models for predicting antibiotic exposure in major tissues associated with common infections can be used to deduce antibiotic efficacy at the site of infection. Additional case reports are essential in achieving this aim (Wu et al.).

In conclusion, all solutions implemented to combat multidrug resistance are double-edged swords because the rate of antibiotic resistance of microorganisms keeps up with the rate of drug progress. Bacteria develop additional mechanisms of resistance in parallel with the spread of novel antimicrobials, such as cefiderocol, ceftolozane/tazobactam, imipenem/relebactam, sulbactam/durlobactam, cefepime-enmetazobactam, and cefepime-taniborbactam. Bacterial susceptibility rates must be continuously monitored to obtain accurate knowledge of the treatment evolution. However, the question remains as to how long these drugs will be useful (Dan and Tǎlǎpan).

Within this scientific panorama, this Research Topic is very interesting, leading to advanced interpretations and solutions for antimicrobial resistance. To deal with resistant bacteria, new generations of the same antibacterial drugs are made by adding new components to the drug to make it effective, or antibiotics are administered in combination to make the bacteria susceptible to either one of the two (Gaibani et al., 2022). However, strategies for antibiotic combination therapy should consider the potential risk of enhanced toxicity.

The molecular studies of resistance genes play an important role if examining the crucial function of the resistance enzymes, such as extended-spectrum β-lactamases (ESBL), carbapenemases (KPC), metallo-β-lactamases (IMP, VIM, NDM etc) or OXA enzymes (OXA_48 and OXA-23) (Shanta et al., 2024).

**Figure 1:**
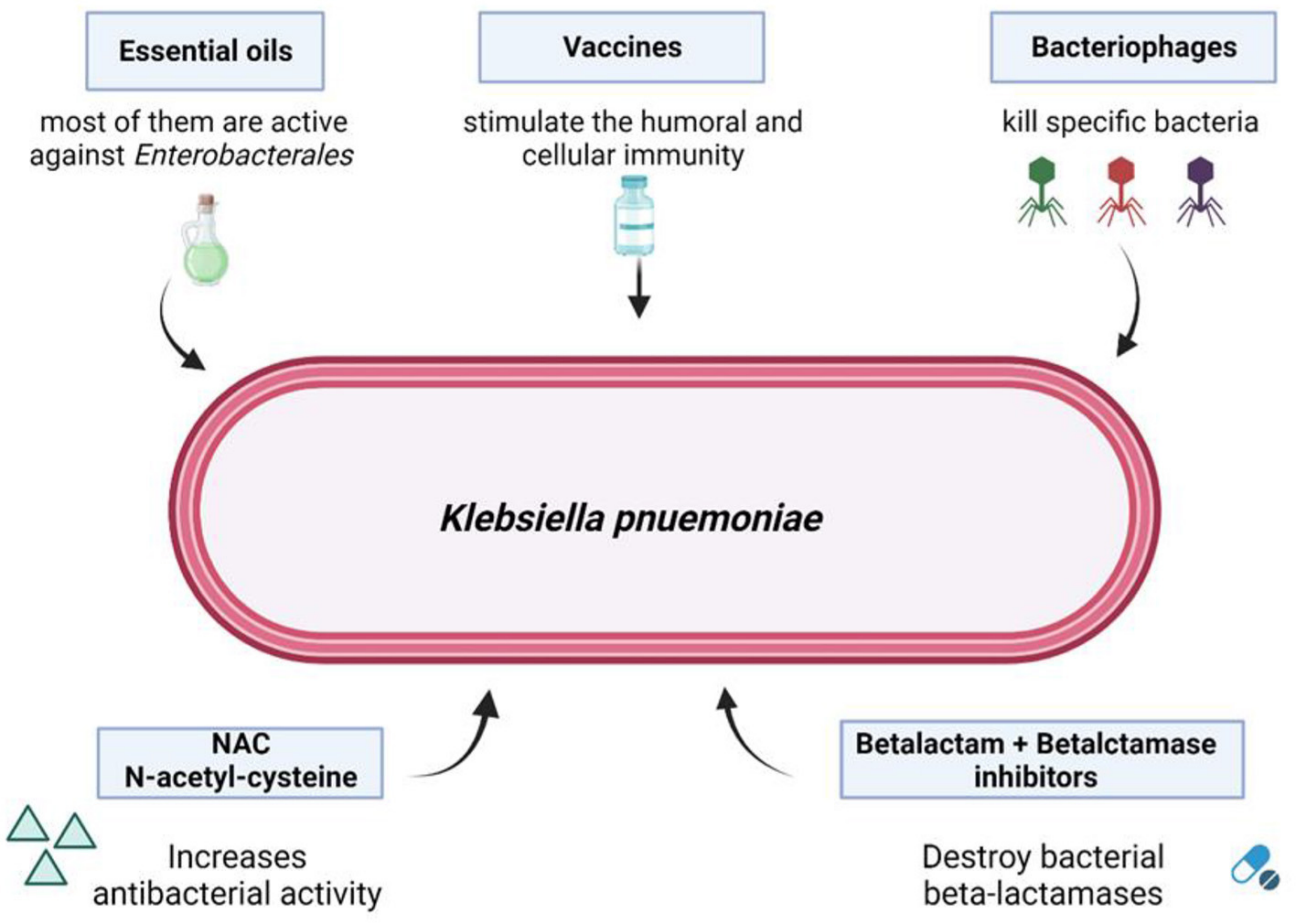
Possible solutions to overcome bacterial carbapenem-resistance. From Mascellino et al. (2024). Source BioRender.com.

In summary, the assessment of resistance mechanisms in common pathogens and the implications of treatment strategies constitute the cornerstones of the fight against resistance. Novel diagnostic techniques, including CRISPR-based technologies (Li et al., 2023), next-generation sequencing and whole genome sequencing should be considered, and different solutions, such as phage therapy (Qin et al., 2021), vaccination strategies, *N*-acethyl-cysteine or the use of conjugated antimicrobial peptides (AMPs) as targeted therapies against pathogenic bacteria responsible for infectious diseases, could be evaluated as alternative treatments for antibiotic-resistant infections (Figure 1).
